# Community Rates of Older Adult Employment in New England: Findings from the 2025 Healthy Aging Data Reports

**DOI:** 10.1093/geroni/igaf122.2499

**Published:** 2025-12-31

**Authors:** Shan Qu, Taylor Jansen, Qian Song, Nina Silverstein, Elizabeth Dugan

**Affiliations:** University of Massachusetts Boston, Boston, Massachusetts, United States; University of Massachusetts Boston, Boston, Massachusetts, United States; University of Massachusetts Boston, Boston, Massachusetts, United States; University of Massachusetts Boston, Boston, Massachusetts, United States; University of Massachusetts Boston, Boston, Massachusetts, United States

## Abstract

Human longevity makes it possible for us to keep working longer, enjoying the relationships and satisfaction of productive activity. However, for some older adults, the high cost of living may require working past age 65 to make ends meet. This study examined and compared community employment rates in four New England states: Rhode Island (RI), Connecticut (CT), New Hampshire (NH), and Massachusetts (MA). Better understanding older adult employment rates at the community level can inform local policies and services. Rates of being employed in the past year among adults 65+ were calculated from data from the 5-year files in the American Community Survey (2012-2016 MA&NH, and 2014-2018 CT&RI). Small area estimation techniques were used to calculate age-sex adjusted community rates. Statewide rates showed that nearly one in four (24.8%) older adults in CT and NH were employed in the past year, which was higher than MA (24.3%) and RI (21.9%). However, MA reported the largest range in 65+ employment (4.5%-63.8%) compared to NH (0.0%-52.2%), CT (14.0%-44.2%), and RI (10.0%-46.3%). Aquinnah MA had the highest community rate in the four states (66.73%). Communities in these four states with the highest rates of older workers reported smaller older populations, lower rates of Hispanic/Latino adults 65+, lower rates living below the poverty line, with higher depression, ADRD, diabetes, and higher median household income (in RI and CT only). The future of work will increasingly include older workers. We think employers should consider flexible work arrangements as inducements to attract and retain older workers.

